# Multicenter evaluation of machine and deep learning methods to predict glaucoma surgical outcomes

**DOI:** 10.3389/frai.2025.1636410

**Published:** 2025-10-22

**Authors:** Samuel Barry, Sophia Y. Wang

**Affiliations:** ^1^Department of Management Science and Engineering, Stanford University, Stanford, CA, United States; ^2^Department of Ophthalmology, Byers Eye Institute, Stanford University, Stanford, CA, United States

**Keywords:** glaucoma, surgical outcome prediction, machine learning, deep learning, electronic health records, multicenter study, clinical prediction models

## Abstract

**Purpose:**

To develop machine learning (ML) and neural network (NN) models to predict glaucoma surgical outcomes, including intraocular pressure (IOP), use of ocular antihypertensive medications, and need for additional glaucoma surgery, using preoperative electronic health records (EHR) from a large multicenter cohort.

**Methods:**

This cohort study included 9,386 patients who underwent glaucoma surgery across 10 institutions in the Sight Outcomes Research Collaborative (SOURCE). All patients had at least 1 year of follow-up and 2 postoperative visits with IOP measurements. Models were trained using preoperative EHR features to predict surgical failure, defined as any of the following: IOP remaining above 80% of preoperative value beyond the immediate postoperative period, increased postoperative glaucoma medications, or need for additional glaucoma surgery. Model performance was evaluated on two test sets: an internal holdout set from sites seen during training and an external holdout set.

**Results:**

Of 13,173 surgeries, 8,743 (66.4%) met failure criteria. The best-performing model for overall surgical failure prediction was a one-dimensional convolutional neural network (1D-CNN) with AUROC of 76.4% and accuracy of 71.6% on the internal test set. The top-performing classical ML model was random forest (AUROC 76.2%, accuracy 72.1%). Prediction performance was highest for IOP-related failure (AUROC 82%), followed by increased medication use (80%) and need for an additional surgery (68%). AUROC declined slightly (2–4%) on the external test set.

**Conclusion:**

ML and DL models can predict glaucoma outcomes using preoperative EHR data. Translational relevance: prediction models may support clinical decision-making by identifying glaucoma patients at risk of poor postoperative outcomes.

## 1 Introduction

Glaucoma is one of the leading causes of blindness worldwide, with prevalence projected to increase by over 50% between 2020 and 2040 (Tham et al., 2014). Patients undergoing glaucoma surgery often have the most severe disease, likely with vision loss that is expected to worsen unless surgery is performed. However, glaucoma surgical outcomes can be highly variable: while surgery can maintain effective disease control over extended periods in some patients with one surgery, other patients may encounter surgical failure at early stages, manifested by inadequate control of intraocular pressure and the need for successive interventions (Wagner et al., 2023). Most previous research investigating predictors of surgical success has considered relatively few and simple patient features, such as age and history of previous surgeries (Hirabayashi et al., 2020; Pantalon et al., 2021; Wagner et al., 2023). However, each patient has a uniquely complex clinical presentation with many factors likely affecting their surgical outcome; this complexity poses significant challenges in predicting post-surgical outcomes with precision. Whether and how long glaucoma surgery is likely to succeed is also likely to depend on the type of glaucoma surgery and how this choice interacts with patient factors.

Previous research leveraging machine learning and deep learning techniques on electronic health records (EHRs) has demonstrated significant potential in predicting various glaucoma-related outcomes, including the probability of glaucoma patients progressing to require surgery and glaucoma surgical outcomes (Jalamangala Shivananjaiah et al., 2023; Tao et al., 2023). One earlier study investigated different prediction model architectures to forecast the success or failure of trabeculectomy surgery at the one-year mark, based on postoperative intraocular pressure (IOP) control, within a relatively small sample of 200 patients (Banna et al., 2022). More recently, another study employed both free-text operative notes and structured EHR data from the preoperative and early postoperative periods to predict IOP outcomes following trabeculectomy in a larger cohort of 1,326 patients (Lin et al., 2024). Finally, our previous study (Barry and Wang, 2024) evaluated machine learning algorithms to predict the outcomes of a wide variety of glaucoma surgical procedures, including trabeculectomy, tube shunts, minimally invasive glaucoma surgeries (MIGS), and cyclodestructive procedures by considering composite failure criterion (IOP control, medication usage, and need for repeat glaucoma surgery). These algorithms outperformed those in prior literature, but several limitations remained, chiefly the single-center nature of the training and testing set.

The goal of the present study is to build upon our previous work by developing and evaluating machine learning and advanced deep learning algorithms to predict outcomes of glaucoma surgery in a large multicenter electronic health records dataset, the Sight Outcomes Research Collaborative (SOURCE) repository.^1^ SOURCE aggregates de-identified EHR from multiple academic eye centers across the U. S. and includes detailed structured information on ocular surgeries and eye examination findings. We continue to employ a composite failure criteria based on intraocular pressure (IOP), glaucoma medication usage, and need for further surgeries, to model surgical outcomes with the greatest possible granularity. We also develop models that predict individual failure criteria as well. The large multicenter cohort drawn from SOURCE also enables external validation of trained models on data from independent sites, additional subgroup analyses, and an assessment of the impact of model training size on the results.

## 2 Methods

### 2.1 Data source and cohort

We identified patients from the SOURCE (Sight Outcomes Research Collaborative) electronic health record database who underwent glaucoma surgery between 2010 and 2022. The SOURCE database collects data from all patients receiving eye care at participating academic health systems, from the time each site implemented the EHR system up to the present (SOURCE Consortium, 2024). This study utilized data from 10 active SOURCE sites, with each site contributing between 4 and 12 years of data. SOURCE includes detailed patient information, such as demographics, diagnoses (based on ICD billing codes), eye examination findings from every clinic visit, and data on medications, laser treatments, and surgical interventions. While the data in SOURCE is fully de-identified, privacy-preserving software (Datavant Inc.) enables researchers to track patients longitudinally across different institutions while safeguarding patient identities.

The glaucoma procedures considered included trabeculectomy and ExPress shunts (CPT codes: 66170, 66172, 66160, 66183), tube shunts (66179, 66180), minimally invasive glaucoma surgery (MIGS: 0191T, 0192T, 66989, 66991, 0253T, 0474T, 0376T, 66174, 66175, 65820, 65850), and cyclophotocoagulation or ciliary body laser procedures (CBL) (66710, 66711, 66720, 66740, 66987, 66988). Patients were included if they had at least two postoperative visits with intraocular pressure (IOP) measurements in the operated eye and 1 year of follow-up. This study was approved by the Stanford University Institutional Review Board and adhered to the principles of the Declaration of Helsinki.

### 2.2 Outcome definition/prediction target

The primary prediction target was glaucoma surgical outcome dichotomized to success/failure, defined as previously described in our original single-center study using multiple criteria incorporating IOP control, glaucoma medication use, and the need for subsequent glaucoma surgery (Barry and Wang, 2024). Briefly, a surgery was considered successful if the postoperative IOP was reduced by more than 20% from baseline, without an increase in glaucoma medications or further glaucoma surgery. The surgery was deemed unsuccessful if any of the following occurred: (1) IOP failure, where the IOP was above 80% of preoperative levels on two consecutive visits beyond the initial 3 months post-surgery; (2) medication failure, where there was an increase in the number of glaucoma medication categories, including carbonic anhydrase inhibitors, beta blockers, alpha agonists, prostaglandins, miotics, oral carbonic anhydrase inhibitors, or rho kinase inhibitors; (3) glaucoma surgery failure, defined as the need for additional glaucoma surgery or revision within 3 months of the original procedure. A non-successful surgery was considered a failure, and vice versa.

As the definition of a successful IOP outcome can vary by patient, surgeon, and type of surgery, models were also developed for alternative IOP failure thresholds, following the World Glaucoma Association Guidelines (Shaarawy et al., 2009): IOP > 12, 15, 18, or 21 mm Hg at two consecutive postoperative visits and IOP above 80% of preoperative IOP at two successive postoperative visits. Thus, potential users of such a model may select the failure definition that best aligns with their desired level of stringency. Outcomes were determined based on EHR data across all sites.

### 2.3 Feature engineering

The feature engineering process was similar to what was previously described for our single-center study (Barry and Wang, 2024). Input features were extracted from electronic health records (EHR), including demographics, past ocular surgeries, diagnoses, medications, social history, and clinical exam findings. Categorical features were one-hot encoded, and continuous variables were standardized (mean = 0, variance = 1). All feature values were collected at baseline, from the preoperative period.

Categorical variables included surgery CPT code, race, ethnicity, gender, prior diagnoses (ICD codes), preoperative medications, prior glaucoma surgeries, concurrent cataract surgery, type of glaucoma surgical implant and/or supply used for the operation (e.g., Ahmed, Baerveldt, Hydrus, Kahook Dual Blade, etc.), and health-related behaviors (e.g., tobacco, alcohol, or drug use). Ocular and systemic medications were recorded as Boolean variables, indicating whether the patient had been prescribed the medication within 5 years before surgery. Variance elimination was performed to retain the 100 features with the highest variance each for systemic medications. ICD codes were aggregated to two decimal places (e.g., H25.011 became H25.01) to reduce the dimensionality of the feature space.

Continuous variables included age, the latest preoperative IOP value, visual acuity (VA), central corneal thickness, refraction spherical equivalent, and the number of prior ophthalmic surgeries. VA was converted to the logarithm of the minimum angle of resolution (logMAR). Continuous variables were standardized, missing value indicator variables were created and missing values were imputed using column means (<7% missingness overall, 0% missingness for IOP). A total of 326 input features were used, including 100 features each for diagnoses, systemic medications, and 28 for ophthalmic medications. To overcome the class imbalance in surgical failure, we leveraged scikitlearn’s SMOTE (Synthetic Minority Over-sampling Technique) method (Chawla et al., 2002), in which synthetic samples of the minority class are artificially generated. We only applied this method to the training data of models predicting surgical failure due to increased medication and the need for follow-up glaucoma surgery as they suffered significant class imbalance.

Data was split for evaluation ensuring that no patient appeared in both training and test sets in the case of multiple surgeries, such as across both eyes. Data from one site comprising 980 patients and 1,499 surgeries was held out as an external test set. This external test set was drawn from a single clinical site not represented in the training or internal test sets, allowing us to assess the model’s ability to generalize to previously unseen, out-of-distribution data. The remaining data was split between a set used for training and cross-validation (80% of surgeries, *N* = 9,339) and an internal test set (20% of surgeries, *N* = 2,335) meant to evaluate in-distribution performance.

### 2.4 Modeling approach

All models were trained to predict overall surgical failure and specific failure types (IOP, medication, or need for additional surgery). We trained several classical machine learning models using scikit-learn (v1.1.3) (Pedregosa et al., 2011), including decision trees, random forest, XGBoost, penalized logistic regression, multi-layer perceptron, k-nearest neighbors, Gaussian naive Bayes, linear discriminant analysis, and support vector machines. The hyperparameters for these models, outlined in Supplementary Table S1, were tuned using grid search and five-fold cross-validation on the training set, and the best model was evaluated on the test set. The classification threshold was optimized for accuracy. Two deep learning architectures were also benchmarked: 1-Dimensional Convolutional Neural Networks (1D-CNN) (O’Shea and Nash, 2015; Kiranyaz et al., 2019) and Attentive Interpretable Tabular Learning (TabNet) (Arik and Pfister, 2021). Dropout layers were added to the 1D-CNN model to prevent overfitting and hyperparameters such as the learning rate and the dimension of hidden layers for 1D-CNN and attention mechanism and layer configuration for TabNet were benchmarked (Supplementary Table S1). Early stopping was based on validation loss, with a patience of 10, and the model with optimal classification threshold for accuracy was chosen.

We also investigated the impact of training set size on model performance by training our top models on subsets of the *N* = 9,339 total training size, with the subset size ranging from *N* = 100 to *N* = 9,339. For each subset, the results were averaged over 10 replicates: for each training set size, 10 different randomly sampled subsets of the training population were chosen, and the model was trained on each of these subsets and evaluated on the test set, with results averaged across these 10 replicates.

### 2.5 Evaluation

#### 2.5.1 Standard evaluation metrics

All models were assessed using standard classification metrics, including accuracy, recall, specificity, precision, negative predictive value, and F1 score. Area under the receiver operating curve (AUROC) and precision-recall curve (AUPRC) were also evaluated. Metrics were computed for both the internal and external test sets. Confidence intervals were calculated via clustered bootstrapping (Ying et al., 2022; Huang, 2018) to account for within-patient clustering, as some patients underwent multiple surgeries and thus contributed multiple observations to the dataset. The AUROC and accuracy of the two best-performing models were then evaluated on subsets of the population based on surgery type, race, ethnicity, age, and intraocular pressure. We also evaluated model calibration using Brier scores and calibration curves.

#### 2.5.2 Explainability

We used SHapley Additive exPlanations (SHAP) (Lundberg and Lee, 2017) to interpret feature importance, as previously described (Barry and Wang, 2024). SHAP values quantify both the magnitude and directionality of each feature’s marginal contribution to a model’s prediction. This technique computes the Shapley values for each feature, a concept originally used in game theory to measure the contribution of each player in a cooperative game. Importantly, use of SHAP is not aimed at identifying novel risk factors or causal relationships, but rather to serve as a sanity check of model behavior. In our study, the SHAP TreeExplainer (Lundberg, 2024) was applied to the random forest model, the best-performing non-deep learning model, allowing us to identify the most influential features driving the predictions. SHAP values were computed for both the internal and external test set. In addition, to get an understanding of the most important features for each model, permutation importance was quantified by shuffling individual features and measuring the ΔAUROC, following the statistical framework described by Altmann et al. (2010).

## 3 Results

### 3.1 Population characteristics

**Figure 1:**
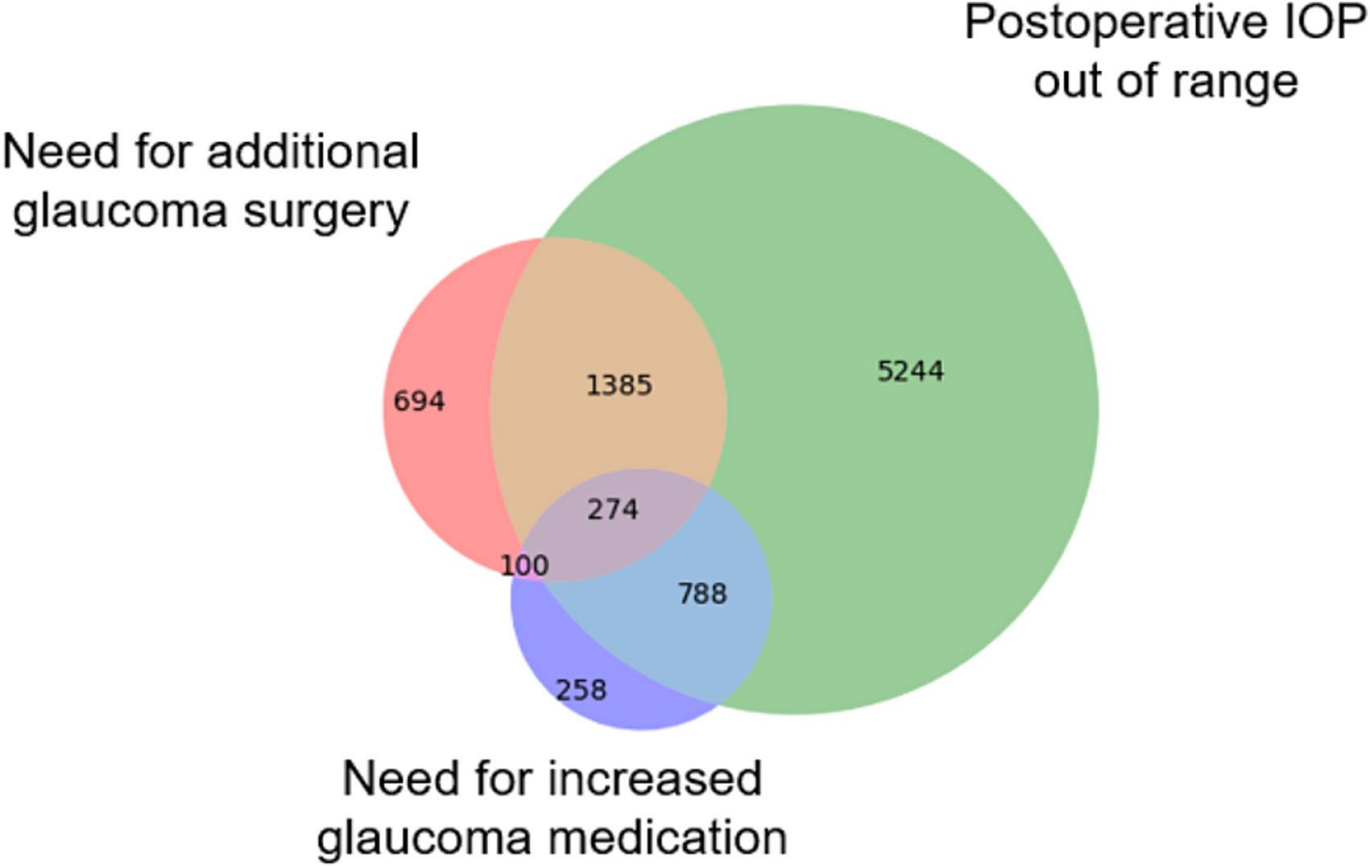
Causes of glaucoma surgical failure. The Venn diagram illustrates the number of surgeries that failed based on three distinct criteria types: (1) IOP failure, defined as a postoperative reduction of <20% compared to preoperative levels; (2) Medication failure, where more classes of glaucoma medications are needed post-surgery than pre-surgery; and (3) Surgical failure, requiring additional glaucoma surgery or revision of the original procedure within three months.

A total of 9,386 patients who underwent 13,173 glaucoma surgeries were included in the overall cohort, including 980 patients and 1,499 operations from one site which was held out as an external test set. The overall surgical success rate was 33.6% (*N* = 4,430), while 66.4% (*N* = 8,743) met the composite failure criteria (Figure 1). Among the three individual failure criteria, IOP failure was the most common (*N* = 7,691 [88.0%]). Failure due to the need for an additional glaucoma surgery or revision was reported in 2453 (28.1%) cases of surgical failure, and 1,420 (16.2%) procedures failed due to medication failure, where the patient requires more classes of ocular antihypertensive medication after surgery than before. Failure rates varied by procedure type: tube shunt (54.3%, 1854/3414), trabeculectomy (62.3%, 2124/3410), cyclophotocoagulation (69.1%, 1722/2492), and MIGS (78.9%, 3043/3857). Failure rates for the alternative IOP success criteria (IOP reduction of 20% or IOP ≤ 12, 15, 18, or 21 mm Hg) ranged from 36.5 to 63.0% for internal and 36.4 to 66.1% for external test set and are shown in Supplementary Table S2.

Table 1 summarizes the population characteristics. The mean age was 65.6 years (SD = 16.6), with 51.3% of the cohort being female (*N* = 4,812). The majority were White (54.1%, *N* = 5,074) or Black (22.2%, *N* = 2083). Preoperative intraocular pressure (IOP) averaged 22.5 mmHg (SD = 10.0), and the mean LogMAR visual acuity was 0.80 (SD = 1.1), roughly equivalent to 20/90 Snellen acuity. The spherical equivalent was −1.16 D (SD = 3.5). Preoperatively, 34.1% of patients had used latanoprost, 25.8% brimonidine, and 21.5% dorzolamide-timolol. Demographic distributions varied across institutions. For example, in the external test site, Asian (30.4%) and Hispanic (16.8%) patients were represented at higher proportions than in the internal training cohort (6.5% Asian, 7.0% Hispanic). Conversely, Black (5.2%) and White (35.0%) patients were proportionally less represented in the external cohort compared with the internal set (24.2% Black, 56.3% White). These differences reflect the demographic heterogeneity of patients across SOURCE sites.

**Table 1:** Population characteristics.

	Total *N* surgeries = 13,173 *N* patients = 9,386		Train/internal test set *N* surgeries = 11,674 *N* patients = 8,407		External test set *N* surgeries = 1,499 *N* patients = 980	
	Mean	Std	Mean	Std	Mean	Std
Age	65.6	16.6	65.5	16.7	66.7	15.5
Preoperative clinical characteristics						
IOP (mmHg)	22.5	10.0	22.7	10.0	21.1	9.3
Central Corneal Thickness (μm)	552.8	63.8	552.4	63.0	555.0	68.8
Refraction (D)	−1.16	3.5	−1.08	3.4	−1.77	4.2
LogMAR VA	0.80	1.1	0.81	1.1	0.79	1.0

Demographics	*N*	%	*N*	%	*N*	%
Gender, Female	4,812	51.3%	4,350	51.7%	463	47.2%
Race, Asian	843	9.0%	545	6.5%	298	30.4%
Race, Black	2083	22.2%	2033	24.2%	51	5.2%
Race, White	5,074	54.1%	4,731	56.3%	343	35.0%
Race, Unknown	167	1.8%	155	1.8%	12	1.2%
Race, Other	1,219	13.0%	943	11.2%	276	28.2%
Ethnicity, Hispanic	751	8.0%	586	7.0%	165	16.8%
Ethnicity, Non-Hispanic	8,459	90.1%	7,659	91.1%	801	81.7%
Ethnicity, Unknown	176	1.9%	162	1.9%	14	1.4%

Pre-operative medication use	*N*	%	*N*	%	*N*	%
Latanoprost	3,203	34.1%	2,705	32.2%	498	50.8%
Brimonidine	2,418	25.8%	1997	23.8%	422	43.1%
Dorzolamide/Timolol	2017	21.5%	1751	20.8%	266	27.1%
Timolol	1,424	15.2%	1,111	13.2%	313	31.9%
Acetazolamide	1,094	11.7%	871	10.4%	223	22.9%
Dorzolamide	1,211	12.9%	1,012	12.0%	199	20.3%

Std, Standard Deviation; LogMAR, Logarithm of the Minimum Angle of Resolution; IOP, Intraocular Pressure.

### 3.2 Machine learning and deep learning model results

**Figure 2:**
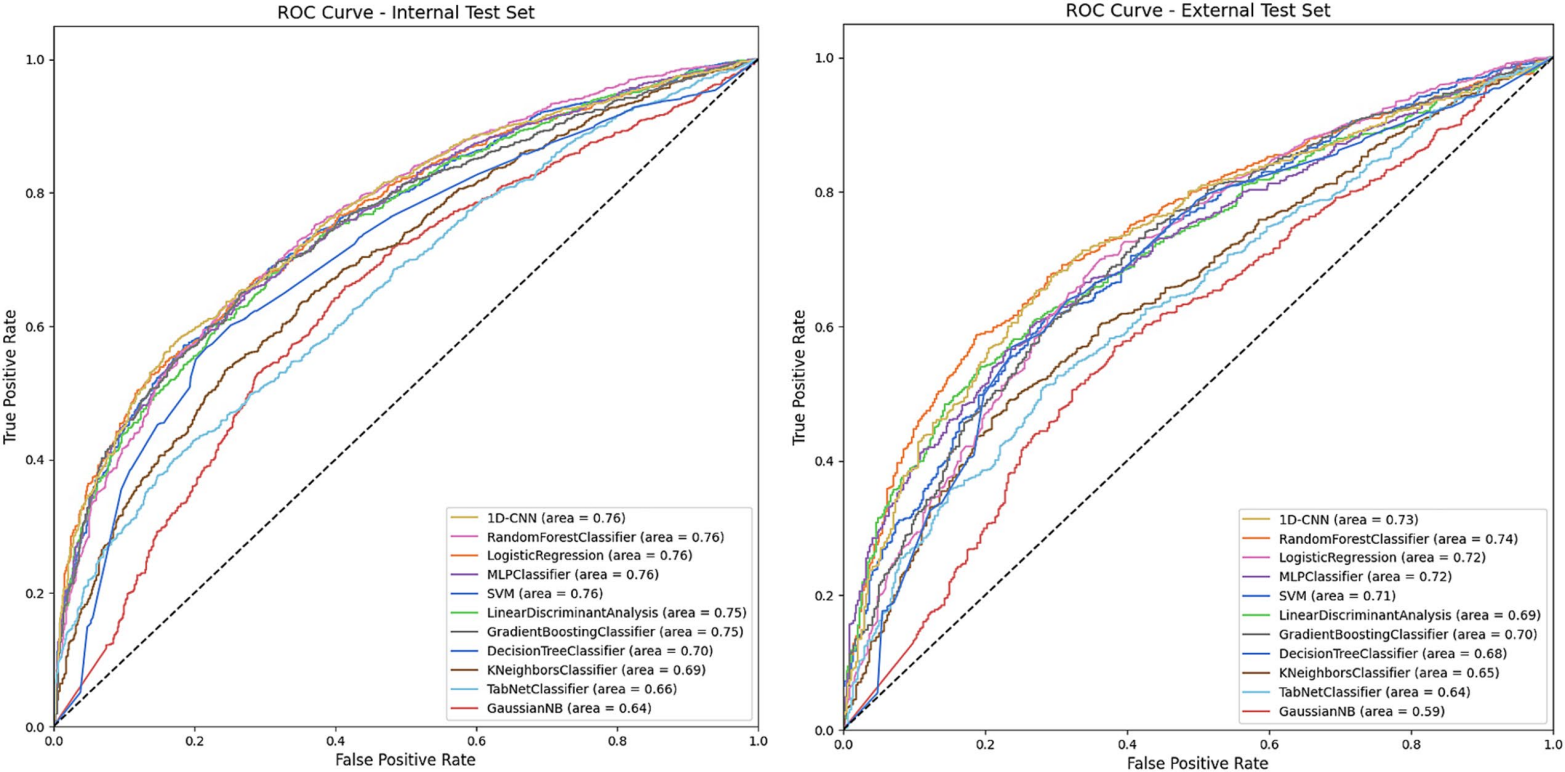
Receiver operating characteristic (ROC) on the internal and external test sets for models predicting overall glaucoma surgical failure. The figures depict the performance of various machine learning and deep learning models in predicting overall glaucoma surgical failure using the internal and external held-out test sets. The legend specifies the model type and the area under the curve (AUC) for each. The models included are Decision Tree, Gradient Boosting, K-Nearest Neighbors, Linear Discriminant Analysis, Logistic Regression, MLP (Multilayer Perceptron), Gaussian Naïve Bayes, Random Forest, SVM (Support Vector Machine), TabNet and 1D-CNN (1-Dimensional Convolutional Neural Network).

We trained a series of machine learning and deep learning models to predict glaucoma surgical failure. Figure 2 depicts the receiver operating characteristic curves for models evaluated on the internal and external test sets; precision-recall curves are displayed in Supplementary Figure 1. Table 2 shows the classification performance metrics. The model with the highest AUROC on the internal test set was 1D-CNN (0.764, 95% CI 0.748–0.778), followed by random forest which also scored the highest accuracy and F1 score (AUROC = 0.762, accuracy = 0.721, F1 = 0.806). The remainder of the models’ AUROC ranged between 0.637–0.761. The random forest model demonstrated superior generalizability, achieving the highest performance in the external test set with AUROC = 0.744. The majority of the models exhibited a slight decrease in performance upon external evaluation, with a loss of approximately 0.03–0.04 in AUROC from the internal to the external test set. Calibration curves and Brier scores are shown in Supplementary Figure 2 and indicate that models were well-calibrated relative to their AUROC performance.

**Table 2:** Model performance for prediction of overall glaucoma surgical failure.

Internal test set							
Model	AUROC (95% CI)	Accuracy (95% CI)	F1 (95% CI)	Sensitivity (recall) (95% CI)	Specificity (95% CI)	PPV (precision) (95% CI)	NPV (95% CI)
1D-CNN	**0.764** (0.748–0.778)	0.716 (0.701–0.733)	0.794 (0.780–0.807)	0.814 (0.801–0.832)	0.517 (0.487–0.542)	0.773 (0.753–0.790)	0.581 (0.555–0.611)
Random Forest	0.762 (0.746–0.775)	**0.721** (0.703–0.733)	**0.806** (0.790–0.816)	**0.863** (0.847–0.877)	0.432 (0.405–0.456)	0.754 (0.737–0.767)	**0.610** (0.582–0.638)
Logistic Regression	0.761 (0.744–0.775)	0.711 (0.695–0.724)	0.786 (0.772–0.797)	0.795 (0.780–0.811)	0.541 (0.518–0.562)	0.778 (0.761–0.791)	0.568 (0.538–0.588)
Multi-Layer Perceptron	0.760 (0.743–0.773)	0.709 (0.695–0.722)	0.786 (0.773–0.796)	0.797 (0.784–0.813)	0.529 (0.504–0.552)	0.774 (0.759–0.788)	0.566 (0.540–0.592)
SVM	0.758 (0.741–0.769)	0.705 (0.686–0.718)	0.779 (0.764–0.790)	0.779 (0.760–0.796)	0.553 (0.525–0.575)	0.778 (0.761–0.793)	0.554 (0.525–0.575)
LDA	0.750 (0.735–0.763)	0.709 (0.695–0.723)	0.797 (0.786–0.808)	0.859 (0.847–0.871)	0.406 (0.381–0.431)	0.745 (0.730–0.758)	0.588 (0.556–0.616)
Gradient Boosting	0.749 (0.734–0.764)	0.701 (0.684–0.712)	0.773 (0.756–0.783)	0.760 (0.739–0.773)	0.579 (0.558–0.605)	0.785 (0.768–0.799)	0.546 (0.520–0.566)
Decision Tree	0.703 (0.682–0.717)	0.681 (0.665–0.696)	0.762 (0.746–0.775)	0.761 (0.744–0.778)	0.526 (0.500–0.550)	0.764 (0.745–0.778)	0.522 (0.497–0.542)
KNN	0.691 (0.672–0.708)	0.685 (0.672–0.705)	0.785 (0.775–0.800)	0.859 (0.848–0.873)	0.336 (0.309–0.361)	0.722 (0.708–0.739)	0.541 (0.509–0.585)
Tab Net	0.660 (0.638–0.676)	0.633 (0.612–0.650)	0.722 (0.703–0.736)	0.711 (0.687–0.731)	0.475 (0.442–0.507)	0.733 (0.708–0.747)	0.448 (0.423–0.478)
Gaussian Naïve Bayes	0.637 (0.615–0.659)	0.476 (0.464–0.494)	0.431 (0.406–0.450)	0.296 (0.274–0.312)	**0.846** (0.824–0.866)	**0.794** (0.766–0.819)	0.373 (0.358–0.393)

External test set							
Model	AUROC (95% CI)	Accuracy (95% CI)	F1 (95% CI)	Sensitivity (recall) (95% CI)	Specificity (95% CI)	PPV (precision) (95% CI)	NPV (95% CI)
1D-CNN	0.730 (0.714–0.751)	0.709 (0.692–0.726)	0.796 (0.782–0.809)	0.796 (0.774–0.810)	0.498 (0.461–0.535)	0.797 (0.779–0.815)	0.498 (0.455–0.530)
Random Forest	**0.744** (0.727–0.765)	**0.736** (0.722–0.751)	**0.826** (0.816–0.836)	**0.881** (0.868–0.895)	0.384 (0.347–0.413)	0.779 (0.763–0.791)	**0.565** (0.517–0.605)
Logistic Regression	0.721 (0.699–0.742)	0.716 (0.700–0.734)	0.802 (0.789–0.815)	0.809 (0.793–0.824)	0.484 (0.452–0.528)	0.795 (0.779–0.813)	0.510 (0.467–0.538)
Multi-Layer Perceptron	0.717 (0.696–0.739)	0.688 (0.673–0.709)	0.783 (0.771–0.798)	0.791 (0.773–0.817)	0.430 (0.391–0.476)	0.775 (0.761–0.792)	0.458 (0.418–0.504)
SVM	0.711 (0.691–0.733)	0.697 (0.681–0.733)	0.784 (0.770–0.794)	0.771 (0.748–0.787)	0.514 (0.483–0.546)	0.796 (0.779–0.811)	0.478 (0.442–0.505)
LDA	0.695 (0.678–0.720)	0.709 (0.691–0.724)	0.807 (0.793–0.817)	0.856 (0.837–0.871)	0.341 (0.309–0.379)	0.763 (0.747–0.778)	0.492 (0.453–0.532)
Gradient Boosting	0.699 (0.679–0.720)	0.670 (0.653–0.688)	0.756 (0.741–0.772)	0.718 (0.695–0.739)	**0.557** (0.524–0.590)	**0.799** (0.781–0.813)	0.445 (0.411–0.472)
Decision Tree	0.680 (0.661–0.703)	0.703 (0.690–0.720)	0.792 (0.781–0.804)	0.793 (0.778–0.813)	0.482 (0.447–0.520)	0.790 (0.773–0.808)	0.489 (0.449–0.522)
KNN	0.653 (0.637–0.673)	0.689 (0.675–0.704)	0.798 (0.787–0.810)	0.867 (0.853–0.882)	0.251 (0.220–0.285)	0.740 (0.725–0.757)	0.434 (0.388–0.474)
Tab Net	0.636 (0.610–0.660)	0.640 (0.623–0.657)	0.743 (0.731–0.759)	0.732 (0.718–0.761)	0.412 (0.370–0.444)	0.754 (0.738–0.772)	0.385 (0.351–0.417)
Gaussian Naïve Bayes	0.591 (0.565–0.623)	0.473 (0.453–0.500)	0.486 (0.461–0.520)	0.353 (0.326–0.385)	**0.769** (0.742–0.800)	0.791 (0.771–0.815)	0.326 (0.303–0.345)

CI, Confidence Interval; AUROC, Area Under the Receiver Operator Curve; SVM, Support Vector Machine; LDA, Linear Discriminant Analysis; KNN, K-Nearest Neighbors; TabNet, Attentive Interpretable Tabular Learning; CNN, Convolutional Neural Network; PPV, Positive Predictive Value; NPV, Negative Predictive Value. Boldface indicates the best value per column within each dataset (internal/external).

**Figure 3:**
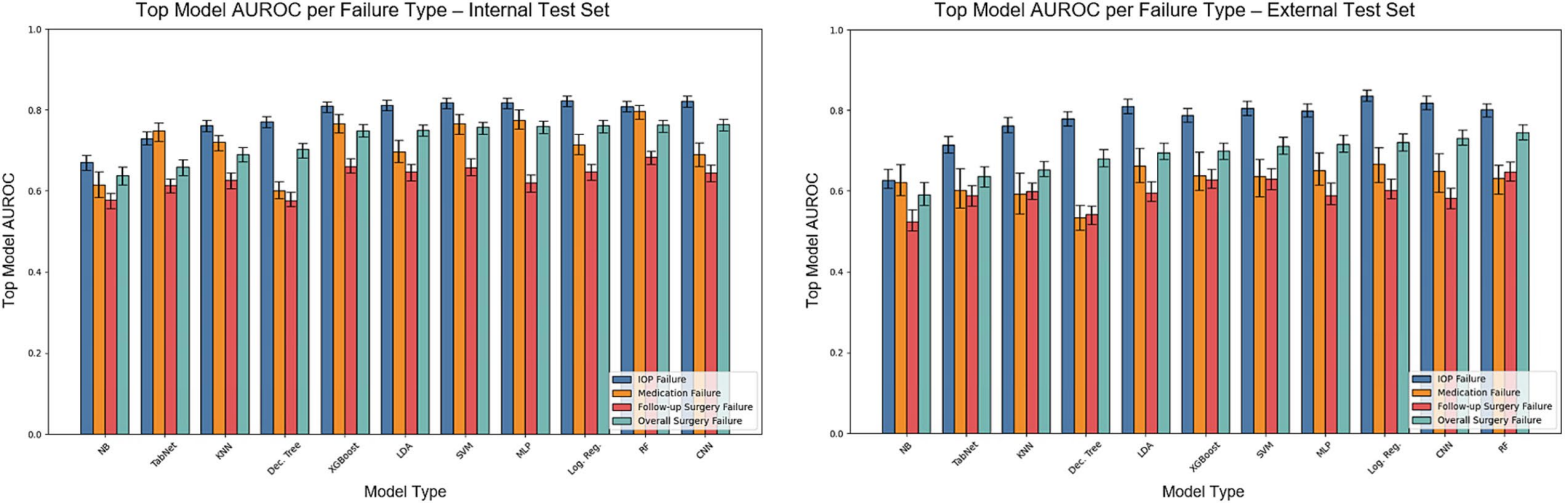
AUROC on the internal and external test sets for models predicting overall surgical failure and specific failure criteria. The bars illustrate the test set AUROC for each model based on individual failure criteria, utilizing the optimal set of hyperparameters. Error bars indicate the 95% confidence intervals. The models included are Dec. Tree (Decision Tree), XGBoost (Gradient Boosting), KNN (K-Nearest Neighbors), LDA (Linear Discriminant Analysis), Log. Reg. (Logistic Regression), MLP (Multilayer Perceptron), NB (Gaussian Naïve Bayes), RF (Random Forest), SVM (Support Vector Machine), TabNet and 1D-CNN (1-Dimensional Convolutional Neural Network).

Figure 3 shows the AUROC scores of the models for each individual surgical failure criterion, by IOP, glaucoma medication usage, or need for follow-up glaucoma surgery. Supplementary Figure 3 also depicts model accuracy for each individual failure criterion. Logistic regression achieved the best performance for failure based on IOP (AUROC = 0.823) whereas random forest outperformed other models to predict medication failure (AUROC = 0.797) and failure due to an additional glaucoma surgery (AUROC = 0.684). Additional model classification performance metrics including recall, precision, F1, and others for predicting individual failure criteria are detailed in Supplementary Tables S3–S5. Results for overall surgical failure, based on alternative IOP thresholds, are provided in Supplementary Table S6, with AUROC values ranging from 0.652 to 0.722 for overall failure with evaluation on the internal test set, and 0.590 to 0.649 for evaluation on the external test set.

Supplementary Table S7 presents an evaluation of the two best-performing models (1D-CNN and random forest) on subsets of the population based on surgery type, race, ethnicity, age, and intraocular pressure. The two models demonstrated stable performance across different population categories, with subgroup AUROC variability often under 5%.

### 3.3 Impact of training set size on model performance

**Figure 4:**
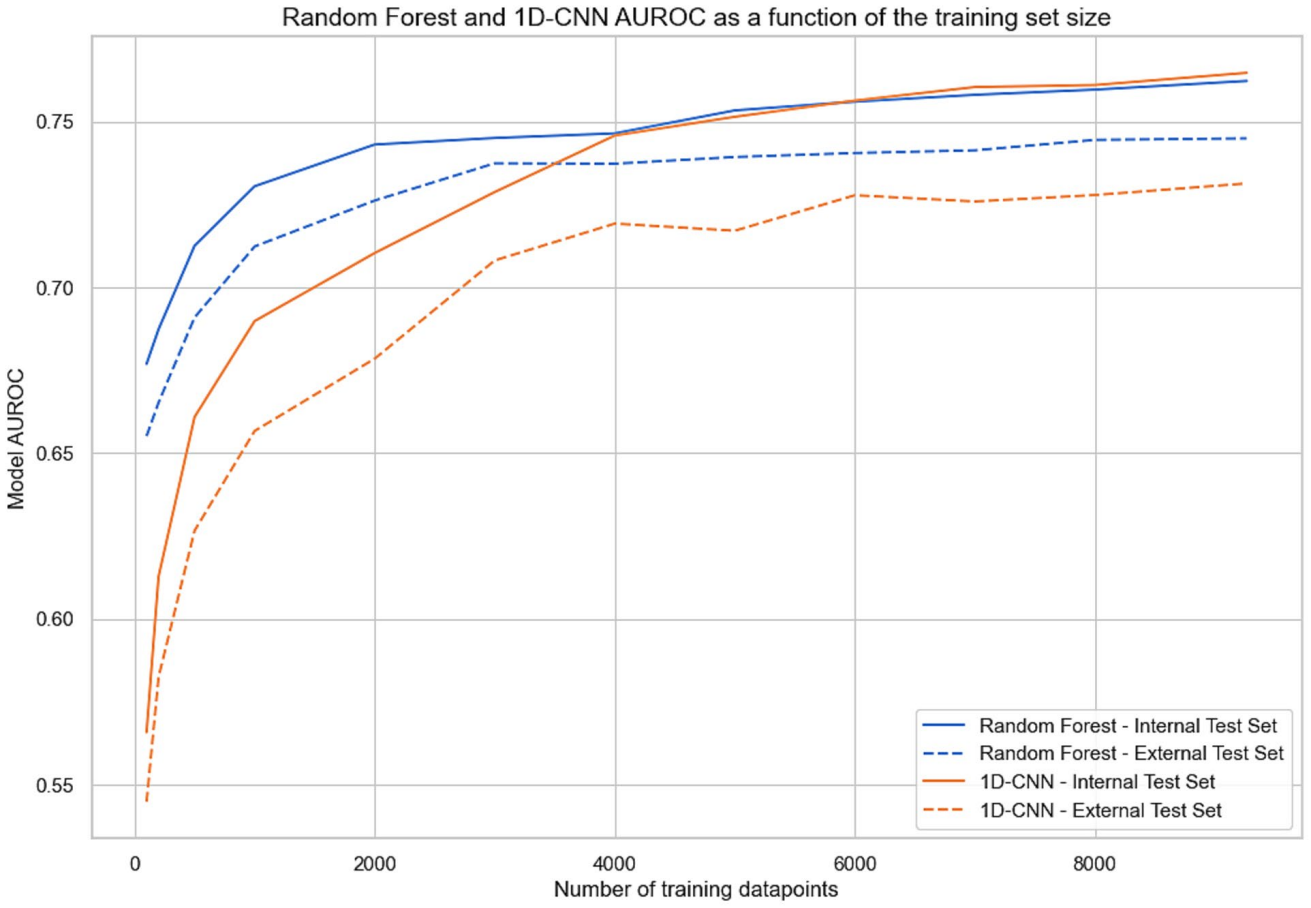
AUROC for random forest and 1D-CNN (1-Dimensional Convolutional Neural Network) evaluated on the internal and external test set as a function of the training set size. For each abscissa N, the two models were trained with a subset of N points from the training set and evaluated on both test sets.

Figure 4 presents the performance of the 1D-CNN and random forest models, trained on varying dataset sizes. Results show the impact of increasing training set size, from *N* = 100 to the full *N* = 9,339 training cohort. The random forest algorithm demonstrated remarkable efficacy in learning from small datasets (e.g., AUROC = 0.68 on the internal test set for *N* = 100 vs. 0.57 for 1D-CNN). However, the 1D-CNN algorithm quickly attained parity with the random forest algorithm as the scale of the training data expanded, outperforming the random forest model on internal test AUROC for a training set of >6,000 training points. On the external test set, random forest always outperformed 1D-CNN at all training set sizes.

### 3.4 Explainability

**Figure 5:**
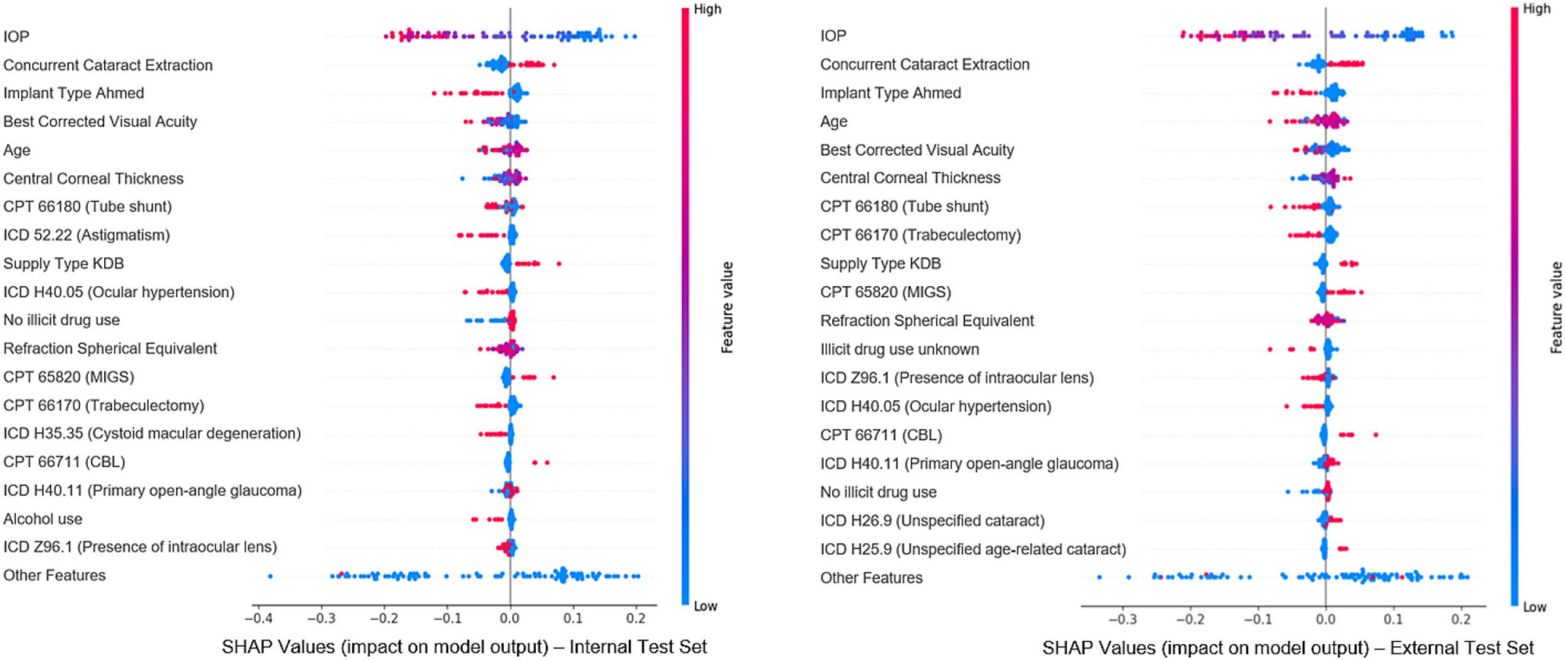
Most important features for model prediction using Shapley analysis. This figure illustrates the Shapley values for the top 20 features deemed most significant in predicting surgical outcomes, based on the best-performing classical machine learning model (random forest). Each dot represents an individual from the cohort, with features listed vertically on the Y-axis and ranked by their importance in the predictive model. The X-axis reflects each feature’s impact on the model’s predictions: values near 0 indicate minimal impact, while values further left or right signify negative or positive effects, respectively. The color of each dot indicates the actual value of that feature for the corresponding individual data point (blue represents low feature values and red represents high feature values).

To evaluate feature importance in predicting surgical outcomes, Shapley values were calculated using the random forest model, the best-performing structured model for overall failure prediction, on both the internal and external test set (Figure 5). The goal of the explainability analysis is not to identify novel risk factors, for which a traditional statistical inference model is better suited. Instead, explainability analyses seek to understand the features the model relies on and determine whether they seem justifiable or not. Features with higher absolute Shapley values had a greater impact on predictions: positive values indicated an association with failure, and negative values indicated surgical success. Clinically relevant features such as IOP, visual acuity, spherical equivalent, concurrent cataract extraction, and surgery type were among the top 20 most important features, demonstrating that the model relies on a variety of reasonable clinical parameters. Additionally, we note that 17 of the top 20 most important features were shared across internal and external test set Shapley values, indicating notable cross-site stability. Supplementary Table 8 reports each model’s top five features via permutation importance. Notably, IOP was the most important feature for all of the 11 models tested, and relevant clinical features such as surgery type, concurrent cataract extraction, age and spherical equivalent were very often part of the top 5.

## 4 Discussion

In this study, we used a large multicenter repository of electronic health records to develop algorithms predicting outcomes of glaucoma surgery. Our novel dataset included diverse types of filtering and minimally invasive glaucoma surgeries and predicted outcomes encompassing a wide range of criteria including intraocular pressure range, use of glaucoma medications, and the necessity for additional glaucoma surgeries. This approach offers unique flexibility in model application for future clinical decision support systems. The large multicenter cohort also enabled the unique and important ability to evaluate the models’ generalizability by reserving data from one site as an external test set. The highest-performing model for predicting overall surgical failure was a 1D-CNN architecture, while the random forest emerged as the top-performing classical machine-learning algorithm. Using only preoperative structured EHR data available in a real-world clinical context, several algorithms achieved an area under the receiver operating characteristic (AUROC) curve exceeding 0.75 for predicting overall composite surgical outcomes, with prediction of individual outcomes (IOP, glaucoma medication usage, need for reoperation) sometimes exceeding AUROC of 0.8. Although these AUROC results may not yet be sufficient for clinical application, they remain highly promising. First, they represent a significant advancement in the field, as this level of performance has never been reported before on such a large dataset - especially with an external holdout set. Second, the task itself is inherently difficult: predicting the future success of a surgery is a challenge beyond standard diagnosis or classification tasks; human baseline performance in predicting future glaucoma outcomes is low (Hu and Wang, 2022). Given the modest performance differences between CNNs and simpler approaches, the choice of model in practice may hinge more on interpretability and ease of implementation, with models such as logistic regression and random forests offering clearer insights for clinicians, while CNNs may prove advantageous as data volume and heterogeneity increase.

Our models predicting outcomes of glaucoma surgery were based on an exceptionally large and diverse set of glaucoma surgeries from multiple centers across the US. Prior studies that have developed prediction algorithms for glaucoma surgeries were generally limited to only trabeculectomies (Banna et al., 2022), included postoperative data in the prediction model (Lin et al., 2024), and/or were limited to smaller single-center cohorts (Banna et al., 2022; Lin et al., 2024; Wang et al., 2022a). In contrast, our models, using only preoperative data in the SOURCE repository, outperformed previous approaches in predicting the outcomes of many types of glaucoma surgeries, including filtering surgeries, minimally invasive glaucoma surgeries (MIGS), and ciliary body destructive procedures. Additionally, our approach was unique in developing models capable of predicting multiple types of surgical failure, including several thresholds for defining intraocular pressure (IOP) success. Since defining glaucoma surgical success can vary across the type of surgery and individual patient factors, such a flexible approach may ultimately be needed to maximize the clinical usefulness of predictions. Our primary analysis reports results using a strict IOP success definition - postoperative IOP reduction of > 20%, yielding a relatively low success rate of 33.6% in our cohort. To accommodate a range of definitions of surgical success, we also modeled success as >20% reduction or postoperative IOP < 21 mmHg, for which the success rate was 63.0% on the internal and 66.1% on the external test set - more in line with typical clinical expectations. Even with the increased complexity of our prediction framework, our 1D-CNN model outperformed prior models developed in related studies (which achieved AUROCs in the 0.70–0.75 range) (Banna et al., 2022; Lin et al., 2024; Wang et al., 2022a) reaching an AUROC of 0.764, and on par with our top model from our previous single-center study (Barry and Wang, 2024).

Another unique advantage of the SOURCE repository is that with a large multicenter cohort, subgroup analyses and external site validation could be performed. Overall, our models scored an AUROC approximately 3% lower on the external test set compared to the internal test set for overall surgical failure prediction; with most models demonstrating an AUROC above 0.7 on a completely new site and distribution. The ability for EHR algorithms to generalize across sites is rarely able to be demonstrated, but this result is similar to our previously reported generalizability results on a different EHR algorithm in SOURCE predicting glaucoma patients’ progression to surgery, where algorithms also exhibited a 2–3% drop in performance on external validation (Wang et al., 2023). Despite being highly diverse in location and patient population, SOURCE sites do share the same underlying EHR system and are all academic centers, which may enhance the generalizability of algorithms across sites. The size of the SOURCE dataset also enabled us to evaluate our top-performing models on population subgroups based on surgery type, race, ethnicity, IOP, and age for both the internal and external test sets. Some performance variability was observed across categories of age and IOP as these were features that highly influence outcome prediction, but in general our models appeared to have reasonably similar performance across race/ethnic groups. However, demographic differences across SOURCE sites may still affect model calibration and transportability, underscoring the importance of testing algorithms across diverse populations. This aspect is a key component for the deployment of real-world clinical decision tools since fairness and bias in artificial intelligence remain key topics of discussion and progress (Ravindranath et al., 2025).

In addition to generalizability across sites and subgroups, explainability analyses may also increase user confidence in model predictions by identifying the most significant factors contributing to model predictions. Our explainability analysis revealed that preoperative intraocular pressure (IOP), visual acuity, age, and type of surgery were among the key predictive features. Reassuringly, explainability analyses demonstrated relatively stable feature importance when evaluated across sites and model architectures, indicating that models were relying upon similar features to predict the outcome. These findings provide reassurance that the model relies upon clinically important factors, several of which have previously been associated with outcomes of trabeculectomy and other glaucoma surgeries (Landers et al., 2012; Edmunds et al., 2004; Fontana et al., 2006; Chiu et al., 2022; Issa de Fendi et al., 2016). It is important to note that explainability analyses are not meant to identify novel risk predictors for outcomes, for which traditional statistical analyses are better suited. Caution must accompany the impulse to extend explainability studies beyond model “sanity checking,” as these are not designed to provide causal insights or clinically meaningful conclusions. Rather, understanding which features may contribute to predictions is one method of establishing model trustworthiness.

Several limitations of our study are inherent to the use of EHR and SOURCE data. For example, some patients may have undergone previous ophthalmic surgeries not captured in our electronic health records (EHR) if they were referred from a site outside of SOURCE. Similarly, some patients may have sought follow-up care or surgeries at external facilities. Potential inaccuracies in coding or medication records, particularly in cases where patients were verbally instructed by their physician to discontinue medications without a corresponding update in the EHR could also affect our model training (Hersh et al., 2013). Limitations in medication records may be mitigated by the fact that new medication prescriptions typically require electronic orders, which are captured in the EHR. In light of these limitations, a surgery was considered to meet failure criteria if postoperative glaucoma medication use exceeded preoperative levels at any point, which may represent a conservative estimate of surgical success. Additionally, because preoperative features, such as prior medication plans, diagnoses, surgeries, and ophthalmic constants, were summarized in the feature engineering process, the temporal nature of the preoperative data was not well-represented. Future research could explore the development of new model architectures capable of incorporating the temporal aspects of EHR data to capture the evolution of patients’ conditions over time. This task remains a challenge in the field, as patient histories are highly variable and irregularly sampled, making harmonized sequence modeling difficult. Lastly, our analysis was limited to structured data and did not incorporate image or text data, as SOURCE is currently working toward including these additional modalities, which are not yet available in most patients. There is considerable potential to enhance the predictive accuracy of these models by including imaging data, such as optical coherence tomography, fundus photography, or visual field results. Multi-institutional data sharing of these modalities of data in a standardized manner is an ongoing challenge in our field, but holds great promise for enhancing the development of prediction algorithms in ophthalmology. Incorporating free-text clinical notes using advanced NLP techniques, such as transformer models or long short-term memory (LSTM) architectures (Vaswani et al., 2017; Hochreiter and Schmidhuber, 1997) could be a promising direction, as several previous studies have demonstrated the significant predictive power of these approaches in ophthalmic tasks (Wang et al., 2022b; Hu and Wang, 2022; Peissig et al., 2012). Information from free-text operative notes could provide more granular information about surgical technique which is otherwise unavailable. Finally, these models were trained and evaluated on retrospective observational data, as is common practice for developing initial models. Future validation studies should incorporate a prospective, real-time “silent” EHR deployment to measure calibration drift, workflow fit and decision impact before any clinician-facing use. Once models are validated prospectively, deployment of models into a clinical decision support tool is likely to be a further challenge, particularly as institutions have varied IT infrastructures. However, a potential clinical decision support tool design might include ingestion of patient EHR data upon physician request in the EHR front-end, and display for the physician the success probabilities for different types of glaucoma surgeries.

## 5 Conclusion

In conclusion, we developed machine and deep learning models to predict glaucoma surgery outcomes in a large multi-institutional cohort using preoperative electronic health records data. We showed that 1D-CNN and random forest were the best-performing algorithms for predicting overall surgery failure. We assessed our models on an external test set and subgroups of the population, demonstrating performance consistency of our algorithms across different populations. Future research to improve prediction performance may explore the inclusion of text and imaging data into a multimodal glaucoma surgery prediction model, especially as these modalities become more widely available and shared across institutions. Such algorithms predicting glaucoma surgical outcomes may one day form the basis of future clinical applications to aid glaucoma surgeons in personalizing treatment choices for patients.

## Data Availability

The data analyzed in this study is subject to the following licenses/restrictions: data from the SOURCE consortium is available to researchers affiliated with participating SOURCE sites, upon application. Requests to access these datasets should be directed to www.sourcecollaborative.org.

## Footnotes

^1^ sourcecollaborative.org

## Group member of SOURCE Consortium

Members of the SOURCE Consortium and their site PIs include: Henry Ford Health System: Sejal Amin, Paul A. Edwards; Johns Hopkins University: Divya Srikumaran, Fasika Woreta; Montefiore Medical Center: Jeffrey S. Schultz, Anurag Shrivastava; Medical College of Wisconsin: Baseer Ahmad; Northwestern University: Dustin D. French, Paul Bryar; Scheie Eye Institute: Brian L. Vanderbeek; Stanford University: Suzann Pershing, Sophia Y. Wang; University of Colorado: Jenna Patnaik; Prem Subramanian; University of Maryland: Saleha Munir, Wuqaas Munir; University of Michigan: Joshua Stein, Lindsey DeLott; University of Utah: Brian C. Stagg, Barbara Wirostko; University of West Virginia: Brian McMillian; Washington University: Arsham Sheybani; Yale University: Soshian Sarrapour, Kristen Nwanyanwu; Ji Liu; University of California, San Francisco: Michael Deiner, Catherine Sun; University of Texas – Houston: Robert Feldman; University of Rochester: Rajeev Ramachandran; SUNY Upstate: Preethi Ganapathy; Mount Sinai School of Medicine: Louis R. Pasquale; Oregon Health Sciences University: Michelle Hribar, Merina Thomas. The SOURCE Data Center is located at the University of Michigan. The Chief Data Officer of SOURCE is Joshua Stein. The Lead Statistician of SOURCE is Chris Andrews. More information about SOURCE is available at: https://www.sourcecollaborative.org/.
