# Supplementary material for: Multicenter evaluation of machine and deep learning methods to predict glaucoma surgical outcomes
Source: Front Artif Intell. 2025 Oct 22;8:1636410. doi: 10.3389/frai.2025.1636410 (PMC12586088; doi:10.3389/frai.2025.1636410)
Supplement: Supplementary file 1 [file Data_Sheet_1.pdf]

Supplementary Table 1: List of hyperparameters benchmarked and optimal value found for all ML and NN models and prediction of overall surgical failure

Method	Hyperparameters	Range of values
**Random Forest**	n estimators	[50, 100, **200**]
	max depth	[10, 20, **50**, null]
	bootstrap	[True, **False**]
	classification threshold	[0.1, 0.2, 0.3, 0.4, **0.5**, 0.6, 0.7, 0.8, 0.9]
**SVM**	kernel	["linear", "poly", "**rbf**", "sigmoid"]
	C (regularization)	[**0.1**, 1, 5]
	classification threshold	[0.1, 0.2, 0.3, 0.4, 0.5, **0.6**, 0.7, 0.8, 0.9]
**Gradient Boosting**	learning rate	[0.01, **0.05**, 0.1]
	subsample	[1, **0.5**, 0.2]
	n estimators	[50, 100, **200**]
	max depth	[10, 20, 50, **null**]
	classification threshold	[0.1, 0.2, 0.3, 0.4, 0.5, 0.6, 0.7, 0.8, **0.9**]
**Gaussian Naïve Bayes**	var smoothing	[1e-10, 1e-9, **1e-8**]
	classification threshold	[**0.1**, 0.2, 0.3, 0.4, 0.5, 0.6, 0.7, 0.8, 0.9]
**Linear Discriminant Analysis**	shrinkage	[**None**, "auto"]
	solver	["svd", "**lsqr**", "eigen"]
	classification threshold	[0.1, 0.2, 0.3, 0.4, **0.5**, 0.6, 0.7, 0.8, 0.9]
**Logistic Regression**	penalty	["**l1**", "l2", None]
	fit intercept	[True, **False**]
	C (regularization)	[0.01, **0.1**, 1]
	classification threshold	[0.1, 0.2, 0.3, 0.4, 0.5, **0.6**, 0.7, 0.8, 0.9]
**KNN**	n neighbors	[3, 5, 7, 11, **15**, 21]
	Weights	["uniform", "**distance**"]
	p (Minkowski metric)	[1, 2, **5**]
	classification threshold	[0.1, 0.2, 0.3, 0.4, **0.5**, 0.6, 0.7, 0.8, 0.9]
**Multi-Layer Perceptron**	hidden layers size	[**10**, 50, 100]
	activation	["identity", "**logistic**", "tanh", "relu"]
	classification threshold	[0.1, 0.2, 0.3, 0.4, **0.5**, 0.6, 0.7, 0.8, 0.9]
**Decision Tree**	max depth	[**10**, 20, 40, 60, 80, 100, null]
	min samples leaf	[**1**, 2, 4]
	min samples split	[**2**, 5, 10]
	classification threshold	[0.1, 0.2, 0.3, 0.4, **0.5**, 0.6, 0.7, 0.8, 0.9]
**TabNet**	prediction layer width	[**8**, 32]
	attention embedding width	[**8**, 32]
	number of steps	[2, **5**]
	number of independent GLU	[**2**, 5]
	number of shared GLU	[**2**, 5]

	grouped features	[disabled, **grouped per feature type**]
	classification threshold	[0.1, 0.2, 0.3, 0.4, **0.5**, 0.6, 0.7, 0.8, 0.9]
**1D-CNN**	learning rate	[1e-4, **1e-3**, 1e-2]
	dropout rate	[0, **0.2**, 0.4, 0.6]
	input size multiplier	[**1**, 2, 4]
	classification threshold	[0.1, 0.2, 0.3, 0.4, **0.5**, 0.6, 0.7, 0.8, 0.9]

SVM: Support Vector Machine; KNN: K-Nearest Neighbors; TabNet: Attentive Interpretable Tabular Learning;
CNN: Convolutional Neural Network.

Supplementary Table 2: Number of overall surgery failures and of failure by IOP out of range by type as a function of the IOP failure threshold

Train/Internal test set:

IOP Succes Criterion	Overall Surgery Failures *(%)*	Failures by IOP out of range *(%)*	Failures by IOP out of range, of which …			
			Only IOP failure *(%)*	IOP and med failure *(%)*	IOP and surgical failure *(%)*	IOP, med and surgical failure *(%)*
**IOP reduction of 20% or IOP <= 12mmHg**	7340 *(63.0)*	6396 *(54.9)*	4286 *(36.8)*	708 *(6.1)*	1187 *(10.2)*	233 *(2.0)*
**IOP reduction of 20% or IOP <= 15mmHg**	6320 *(54.3)*	5165 *(44.3)*	3248 *(27.9)*	589 *(5.1)*	1109 *(9.5)*	219 *(1.9)*
**IOP reduction of 20% or IOP <= 18mmHg**	5052 *(43.4)*	3498 *(30.0)*	1980 *(17.0)*	364 *(3.1)*	972 *(8.3)*	182 *(1.6)*
**IOP reduction of 20% or IOP <= 21mmHg**	4250 *(36.5)*	2311 *(19.9)*	1178 *(10.1)*	206 *(1.8)*	772 *(6.6)*	155 *(1.3)*

IOP: Intraocular Pressure.

External test set:

IOP Succes Criterion	Overall Surgery Failures *(%)*	Failures by IOP out of range *(%)*	Failures by IOP out of range, of which …			
			Only IOP failure *(%)*	IOP and med failure *(%)*	IOP and surgical failure *(%)*	IOP, med and surgical failure *(%)*
**IOP reduction of 20% or IOP <= 12mmHg**	991 *(66.1)*	830 *(55.4)*	564 *(37.6)*	53 *(3.5)*	176 *(11.7)*	37 *(2.5)*
**IOP reduction of 20% or IOP <= 15mmHg**	796 *(53.1)*	598 *(39.9)*	369 *(24.6)*	33 *(2.2)*	159 *(10.6)*	37 *(2.5)*
**IOP reduction of 20% or IOP <= 18mmHg**	628 *(41.9)*	382 *(25.5)*	201 *(13.4)*	23 *(1.5)*	125 *(8.3)*	33 *(2.2)*
**IOP reduction of 20% or IOP <= 21mmHg**	545 *(36.4)*	238 *(15.9)*	118 *(7.9)*	9 *(0.6)*	88 *(5.9)*	23 *(1.5)*

IOP: Intraocular Pressure.

Supplementary Table 3: Model performance for prediction of failure due to IOP not meeting the 20% reduction threshold on the internal and external test set

Model	Test Set	AUROC	Accuracy	F1	Sensitivity (Recall)	Specificity	PPV (Precision)	NPV
**Logistic Regression**	Internal	**0.823**	**0.749**	**0.800**	**0.842**	0.614	0.762	**0.728**
	External	**0.836**	**0.766**	**0.833**	**0.884**	0.579	0.768	**0.762**
**1D-CNN**	Internal	0.822	0.744	0.783	0.776	0.701	**0.792**	0.682
	External	0.818	0.745	0.788	0.777	0.693	**0.799**	0.666
**SVM**	Internal	0.818	0.736	0.787	0.822	0.610	0.755	0.701
	External	0.805	0.747	0.805	0.852	0.583	0.762	0.714
**Multi-Layer Perceptron**	Internal	0.817	0.742	0.784	0.788	0.674	0.779	0.686
	External	0.799	0.732	0.781	0.785	0.648	0.777	0.659
**LDA**	Internal	0.811	0.733	0.787	0.833	0.589	0.747	0.707
	External	0.810	0.739	0.795	0.832	0.596	0.762	0.694
**Random Forest**	Internal	0.809	0.740	0.788	0.818	0.626	0.761	0.704
	External	0.802	0.744	0.801	0.852	0.572	0.758	0.712
**Gradient Boosting**	Internal	0.809	0.738	0.784	0.804	0.643	0.766	0.692
	External	0.788	0.734	0.795	0.844	0.560	0.749	0.699
**Decision Tree**	Internal	0.771	0.721	0.770	0.787	0.624	0.753	0.667
	External	0.779	0.737	0.794	0.829	0.594	0.761	0.688
**KNN**	Internal	0.762	0.704	0.764	0.807	0.555	0.725	0.662
	External	0.761	0.708	0.775	0.827	0.521	0.728	0.658
**TabNet**	Internal	0.730	0.678	0.729	0.731	0.598	0.728	0.604
	External	0.714	0.664	0.729	0.737	0.551	0.720	0.571
**Gaussian Naïve Bayes**	Internal	0.671	0.565	0.510	0.384	**0.826**	0.763	0.479
	External	0.626	0.577	0.580	0.482	**0.728**	0.734	0.476

AUROC: Area Under the Receiver Operator Curve; SVM: Support Vector Machine; LDA: Linear Discriminant Analysis; KNN: K-Nearest Neighbors; TabNet: Attentive Interpretable Tabular Learning; CNN: Convolutional Neural Network; PPV: Positive Predictive Value; NPV: Negative Predictive Value.

Supplementary Table 4: Model performance for prediction of surgical failure due to need for a follow-up surgery on the internal and external test set

Model	Test Set	AUROC	Accuracy	F1	Sensitivity (Recall)	Specificity	PPV (Precision)	NPV
**Random Forest**	Internal	**0.684**	0.798	0.172	0.110	0.961	**0.396**	0.820
	External	**0.648**	0.748	0.067	0.037	0.973	0.317	0.760
**Gradient Boosting**	Internal	0.660	**0.800**	0.111	0.065	**0.974**	0.370	0.814
	External	0.628	0.754	0.06	0.033	**0.984**	0.400	0.761
**SVM**	Internal	0.657	0.794	0.175	0.114	0.955	0.372	0.820
	External	0.630	**0.755**	0.117	0.067	0.975	**0.471**	0.765
**Logistic Regression**	Internal	0.648	0.771	0.271	0.222	0.900	0.345	0.829
	External	0.602	0.724	0.240	0.181	0.897	0.359	0.776
**LDA**	Internal	0.647	0.785	0.214	0.153	0.934	0.358	0.823
	External	0.595	0.741	0.198	0.133	0.935	0.397	0.773
**1D-CNN**	Internal	0.644	0.682	**0.345**	0.433	0.742	0.285	**0.846**
	External	0.583	0.629	**0.311**	**0.350**	0.717	0.278	0.775
**KNN**	Internal	0.626	0.699	0.294	0.329	0.786	0.267	0.832
	External	0.600	0.664	0.310	0.310	0.777	0.306	**0.779**
**Multi-Layer Perceptron**	Internal	0.620	0.769	0.207	0.157	0.914	0.299	0.821
	External	0.589	0.728	0.139	0.090	0.931	0.295	0.763
**TabNet**	Internal	0.614	0.745	0.251	0.223	0.869	0.285	0.824
	External	0.590	0.717	0.213	0.159	0.894	0.323	0.769
**Gaussian Naïve Bayes**	Internal	0.578	0.622	0.323	**0.470**	0.658	0.246	0.840
	External	0.525	0.692	0.237	0.201	0.847	0.292	0.769
**Decision Tree**	Internal	0.576	0.734	0.301	0.302	0.837	0.305	0.835
	External	0.541	0.675	0.289	0.273	0.804	0.307	0.776

AUROC: Area Under the Receiver Operator Curve; SVM: Support Vector Machine; LDA: Linear Discriminant Analysis; KNN: K-Nearest Neighbors; TabNet: Attentive Interpretable Tabular Learning; CNN: Convolutional Neural Network; PPV: Positive Predictive Value; NPV: Negative Predictive Value.

Supplementary Table 5: Model performance for prediction of surgical failure due to need for increased glaucoma medication on the internal and external test set

Model	Test Set	AUROC	Accuracy	F1	Sensitivity (Recall)	Specificity	PPV (Precision)	NPV
**Random Forest**	Internal	**0.797**	0.877	0.143	0.089	0.979	0.350	0.893
	External	0.631	0.922	0.000	0.000	0.998	0.000	0.924
**Multi-Layer Perceptron**	Internal	0.775	0.864	**0.309**	0.267	0.940	0.366	0.909
	External	0.651	0.884	0.148	0.130	0.947	0.161	0.928
**Gradient Boosting**	Internal	0.766	**0.885**	0.087	0.049	**0.992**	**0.429**	0.891
	External	0.637	**0.924**	0.000	0.000	**0.999**	0.000	0.924
**SVM**	Internal	0.766	0.868	0.202	0.148	0.961	0.324	0.898
	External	0.636	0.919	0.022	0.012	0.994	0.125	0.924
**TabNet**	Internal	0.748	0.847	0.302	0.291	0.918	0.313	0.911
	External	0.602	0.857	0.123	0.132	0.915	0.116	0.928
**KNN**	Internal	0.721	0.764	0.279	0.405	0.810	0.215	0.913
	External	0.593	0.805	0.181	0.283	0.848	0.131	0.935
**Logistic Regression**	Internal	0.715	0.821	0.274	0.296	0.888	0.252	0.908
	External	**0.667**	0.910	0.107	0.072	0.979	**0.217**	0.928
**LDA**	Internal	0.697	0.816	0.263	0.290	0.884	0.241	0.907
	External	0.662	0.909	0.118	0.079	0.976	0.214	0.928
**1D-CNN**	Internal	0.689	0.664	0.282	**0.580**	0.675	0.186	**0.926**
	External	0.649	0.660	**0.196**	**0.549**	0.669	0.119	**0.948**
**Gaussian Naïve Bayes**	Internal	0.615	0.665	0.253	0.503	0.686	0.169	0.915
	External	0.622	0.797	0.143	0.222	0.844	0.105	0.930
**Decision Tree**	Internal	0.601	0.801	0.280	0.341	0.861	0.239	0.910
	External	0.534	0.759	0.143	0.271	0.797	0.098	0.930

AUROC: Area Under the Receiver Operator Curve; SVM: Support Vector Machine; LDA: Linear Discriminant Analysis; KNN: K-Nearest Neighbors; TabNet: Attentive Interpretable Tabular Learning; CNN: Convolutional Neural Network; PPV: Positive Predictive Value; NPV: Negative Predictive Value.

Supplementary Table 6: Best model performance for prediction of overall glaucoma surgical failure as a function of the IOP failure threshold

IOP Success Criterion	Test Set	AUROC	Accuracy	F1	Sensitivity (Recall)	Specificity	PPV (Precision)	NPV
**IOP reduction of 20% or IOP <= 12mmHg**	Internal	0.722	0.695	0.781	0.852	0.414	0.720	0.613
	External	0.649	0.671	0.778	0.868	0.283	0.703	0.524
**IOP reduction of 20% or IOP <= 15mmHg**	Internal	0.666	0.626	0.689	0.747	0.475	0.640	0.600
	External	0.590	0.565	0.627	0.685	0.426	0.578	0.549
**IOP reduction of 20% or IOP <= 18mmHg**	Internal	0.652	0.611	0.467	0.384	0.796	0.603	0.614
	External	0.633	0.614	0.429	0.348	0.810	0.562	0.632
**IOP reduction of 20% or IOP <= 21mmHg**	Internal	0.653	0.634	0.254	0.164	0.919	0.555	0.645
	External	0.646	0.643	0.240	0.153	0.925	0.535	0.656

AUROC: Area Under the Receiver Operator Curve; PPV: Positive Predictive Value; NPV: Negative Predictive Value; IOP: Intraocular Pressure.

NB: "Best model" defined as best performing model on internal test set AUROC. Results for all metrics/ test sets are reported on this best model for each of the 4 IOP success criterion categories.

Supplementary Table 7: AUROC of the two best performing models (Random Forest, 1D-CNN) on subgroups of the internal and external test set

	**Internal Test Set** N surgeries = 2335		**External Test Set** N surgeries = 1499	
	**RF** *(95% CI)*	**1D-CNN** *(95% CI)*	**RF** *(95% CI)*	**1D-CNN** *(95% CI)*
**Surgery type**				
Trabeculectomy	0.72 *(0.69 - 0.76)*	0.72 *(0.69 - 0.75)*	0.68 *(0.64 - 0.71)*	0.69 *(0.65 - 0.73)*
Tube shunt	0.72 *(0.69 - 0.75)*	0.73 *(0.69 - 0.76)*	0.73 *(0.69 - 0.75)*	0.73 *(0.69 - 0.77)*
Minimal invasive glaucoma surgery	0.79 *(0.75 - 0.82)*	0.77 *(0.73 - 0.81)*	0.69 *(0.64 - 0.74)*	0.73 *(0.69 - 0.78)*
Ciliary Body Laser	0.73 *(0.70 - 0.76)*	0.70 *(0.68 - 0.73)*	0.70 *(0.64 - 0.75)*	0.70 *(0.65 - 0.75)*
**Race**				
Asian	0.76 *(0.71 - 0.81)*	0.75 *(0.69 - 0.80)*	0.73 *(0.68 - 0.77)*	0.72 *(0.68 - 0.76)*
Black	0.77 *(0.74 - 0.80)*	0.77 *(0.74 - 0.81)*	0.78 *(0.68 - 0.89)*	0.82 *(0.77 - 0.87)*
White	0.77 *(0.75 - 0.79)*	0.76 *(0.74 - 0.78)*	0.72 *(0.68 - 0.75)*	0.75 *(0.71 - 0.79)*
Unknown	0.76 *(0.72 - 0.81)*	0.75 *(0.72 - 0.79)*	0.70 *(0.66 - 0.73)*	0.73 *(0.68 - 0.78)*
**Ethnicity**				
Hispanic	0.77 *(0.72 - 0.82)*	0.75 *(0.69 - 0.81)*	0.68 *(0.63 - 0.74)*	0.75 *(0.69 - 0.81)*
Non-Hispanic	0.76 *(0.75 - 0.78)*	0.76 *(0.75 - 0.77)*	0.72 *(0.68 - 0.77)*	0.76 *(0.74 - 0.78)*
Unknown	0.78 *(0.62 - 0.88)*	0.75 *(0.60 - 0.90)*	0.75 *(0.48 - 0.96)*	0.81 *(0.54 - 1.0)*
**Age quartiles**				
1st age quartile (<60 years)	0.72 *(0.69 - 0.75)*	0.72 *(0.65 - 0.79)*	0.67 *(0.64 - 0.72)*	0.69 *(0.65 - 0.74)*
2nd age quartile (>=60, <70 years)	0.80 *(0.77 - 0.83)*	0.79 *(0.76 - 0.82)*	0.77 *(0.72 - 0.81)*	0.79 *(0.74 - 0.83)*
3rd age quartile (>=70, <77 years)	0.75 *(0.72 - 0.78)*	0.75 *(0.72 - 0.78)*	0.69 *(0.64 - 0.74)*	0.73 *(0.69 - 0.78)*
4th age quartile (>=77 years)	0.79 *(0.76 - 0.82)*	0.78 *(0.74 - 0.82)*	0.72 *(0.68 - 0.76)*	0.76 *(0.72 - 0.80)*
**IOP quartiles**				

1st IOP quartile (<15 mmHg)	0.68	0.69	0.59	0.68
	(0.62 - 0.74)	*(0.67 - 0.72)*	*(0.52 - 0.65)*	*(0.59 - 0.75)*
2nd IOP quartile (>=15, <20 mmHg)	0.68	0.65	0.55	0.58
	(0.65 - 0.72)	*(0.61 - 0.67)*	*(0.51 - 0.59)*	*(0.53 - 0.62)*
3rd IOP quartile (>=20, <27 mmHg)	0.65	0.62	0.64	0.64
	(0.62 - 0.68)	*(0.57 - 0.67)*	*(0.60 - 0.69)*	*(0.59 - 0.69)*
4th IOP quartile (>=27 mmHg)	0.61	0.66	0.51	0.46
	(0.58 - 0.65)	*(0.64 - 0.70)*	*(0.46 - 0.56)*	*(0.41 - 0.51)*

RF: Random Forest; CNN: Convolutional Neural Network; AUROC: Area Under the Receiver Operator Curve;IOP: Intraocular Pressure; CI: Confidence Interval

Supplementary Table 8: Top 5 Most Influential Features by Model by Permutation Importance

Rank	1D-CNN	Random Forest	Logistic Regression	MLP	SVM	LDA	Gradient Boosting	Decision Tree	KNN	Tab Net	Gaussian NB
1	IOP	IOP	IOP	IOP	IOP	IOP	IOP	IOP	IOP	IOP	IOP
2	CPT 66170 (Trabeculectomy)	Implant Type Ahmed	CPT 66170 (Trabeculectomy)	CPT 66170 (Trabeculectomy)	Age	CPT 66170 (Trabeculectomy)	CPT 66170 (Trabeculectomy)	CPT 66170 (Trabeculectomy)	Number of past Cornea	Z96.1 (Presence of IOL)	Implant Type iStent
3	Concurrent Cataract Extraction	Concurrent Cataract Extraction	ICD Z01.81 (Pre-operative exam)	Age	CPT 66170 (Trabecu-lectomy)	Implant Type Ahmed	Age	Spherical Equivalent Refraction	CCT	CPT 66711 (CBL)	H40.11 (Open-angle glaucoma)
4	Age	CPT 66170 (Trabeculectomy)	Race: Black	Ill Drug use: No	Implant Type Ahmed	Race: Black	CCT	Implant Type Ahmed	BCVA	Alcohol use: No	Implant Type Ahmed
5	ICD H35.81 (Retinal dystrophy)	CPT 66711 (CBL)	Age	CCT	CCT	ICD Z01.81 (Pre-operative exam	SER	Age	Number of past Shunts	CPT 65820 (Goniotomy)	CPT 0474t (MIGS)

IOP: Intraocular pressure; BCVA: Best Corrected Visual Acuity; SER: Spherical Equivalent Refraction; CCT: Central Corneal Thickness
