# Supplementary material for: Multicenter evaluation of machine and deep learning methods to predict glaucoma surgical outcomes
Source: Front Artif Intell. 2025 Oct 22;8:1636410. doi: 10.3389/frai.2025.1636410 (PMC12586088; doi:10.3389/frai.2025.1636410)
Supplement: Supplementary file 2 [file Data_Sheet_2.pdf]

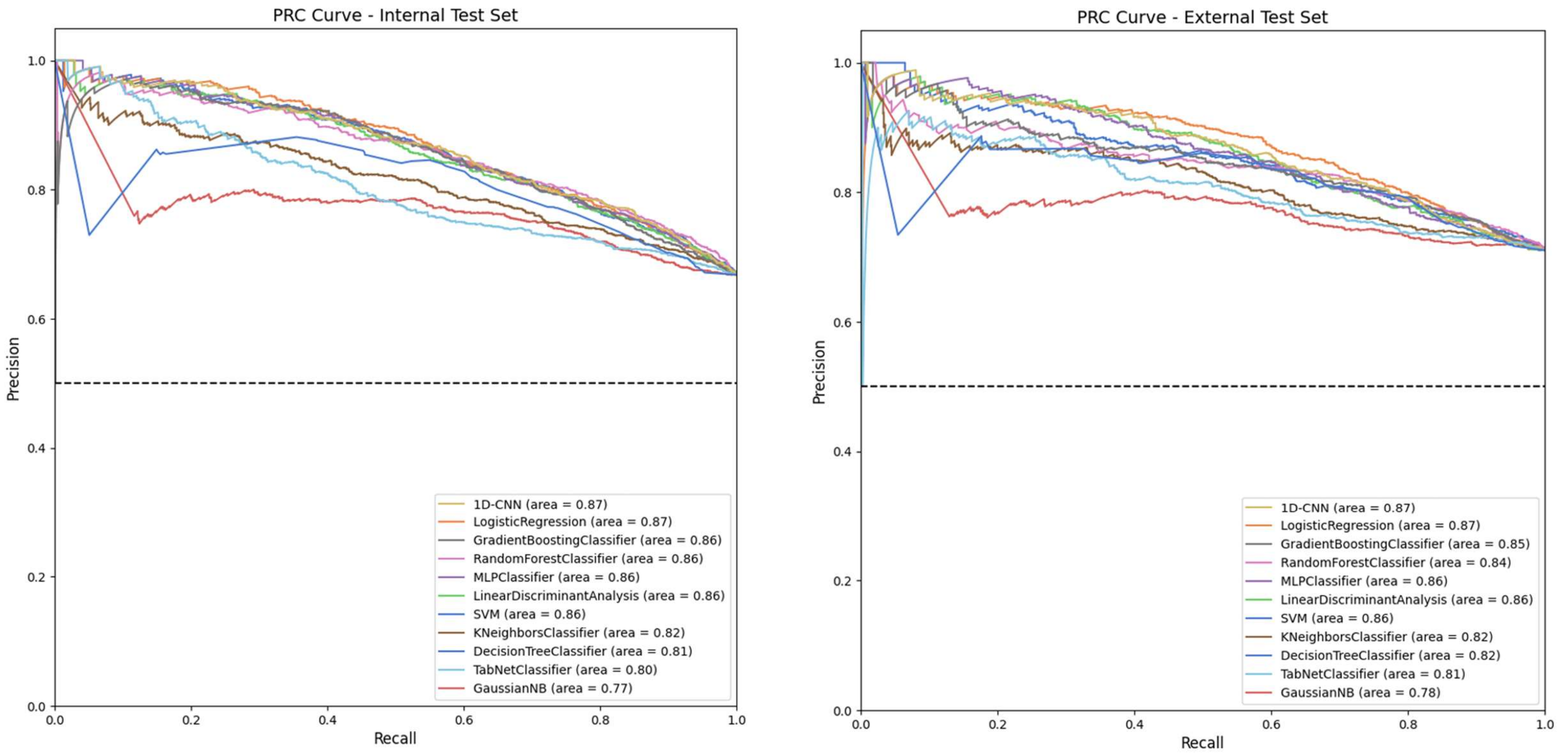

Supplementary Figure 1: Precision-Recall Curve (PRC) on the internal and external test sets for models predicting overall glaucoma surgical failure.
The figures depict the performance of various machine learning and deep learning models in predicting overall glaucoma surgical failure using the internal and external held-out test sets. The legend specifies the model type and the area under the curve (AUC) for each. The models included are Decision Tree, Gradient Boosting, K-Nearest Neighbors, Linear Discriminant Analysis, Logistic Regression, MLP (Multilayer Perceptron), Gaussian Naïve Bayes, Random Forest, SVM (Support Vector Machine), TabNet and 1D-CNN (1-Dimensional Convolutional Neural Network).

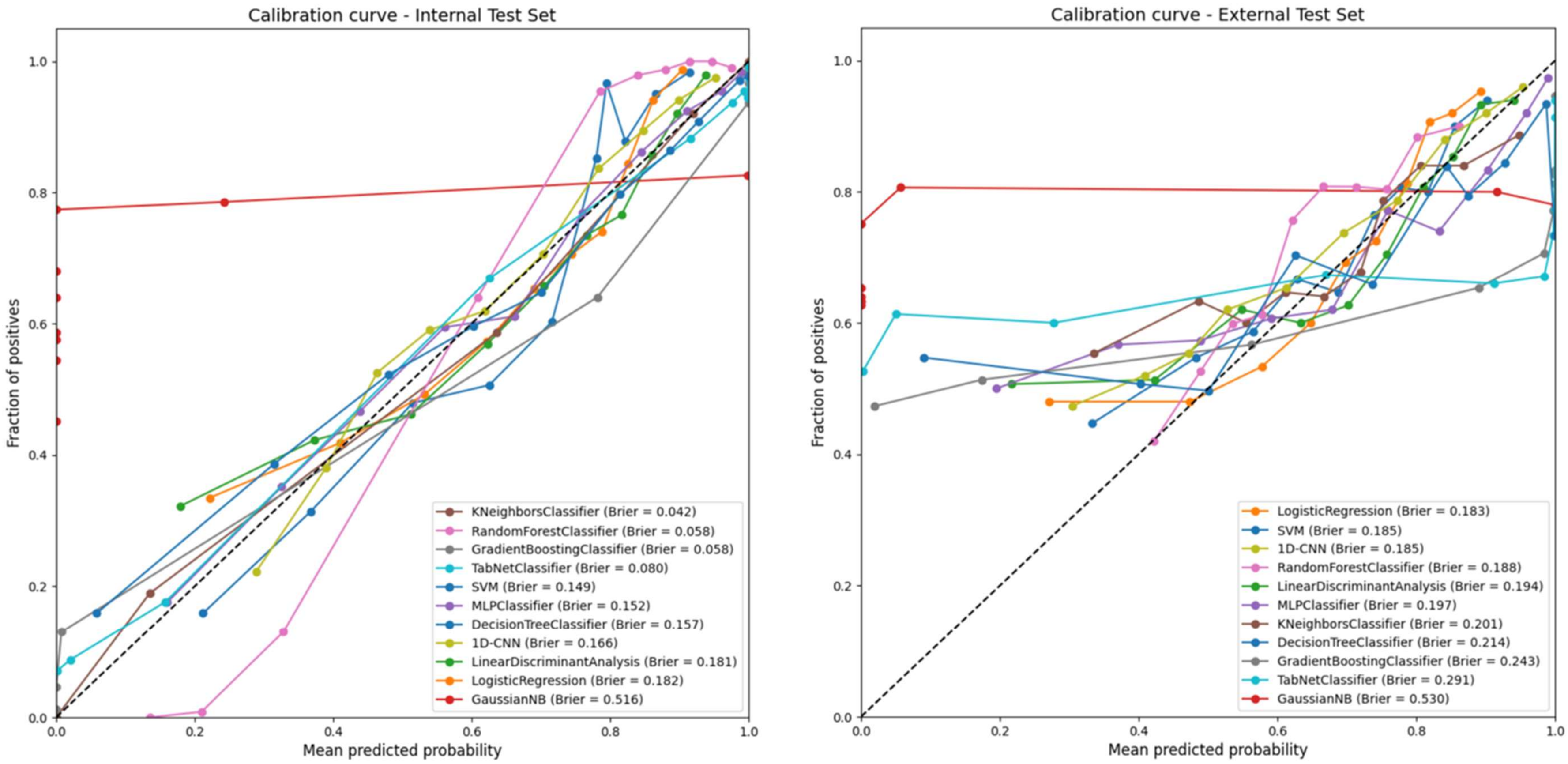

Supplementary Figure 2: Calibration curves on the internal and external test sets for models predicting overall surgical failure.
The connected dotted lines show the calibration curves for each model, for the internal test set (left) and the external test set (Right). The black dashed line indicates perfect calibration. The models included are Decision Tree, Gradient Boosting (XGBoost), KNeighbors (K-Nearest Neighbors), Linear Discriminant Analysis, Logistic Regression, MLP (Multilayer Perceptron), GaussianNB (Gaussian Naïve Bayes), Random Forest, SVM (Support Vector Machine), TabNet and 1D-CNN (1-Dimensional Convolutional Neural Network).

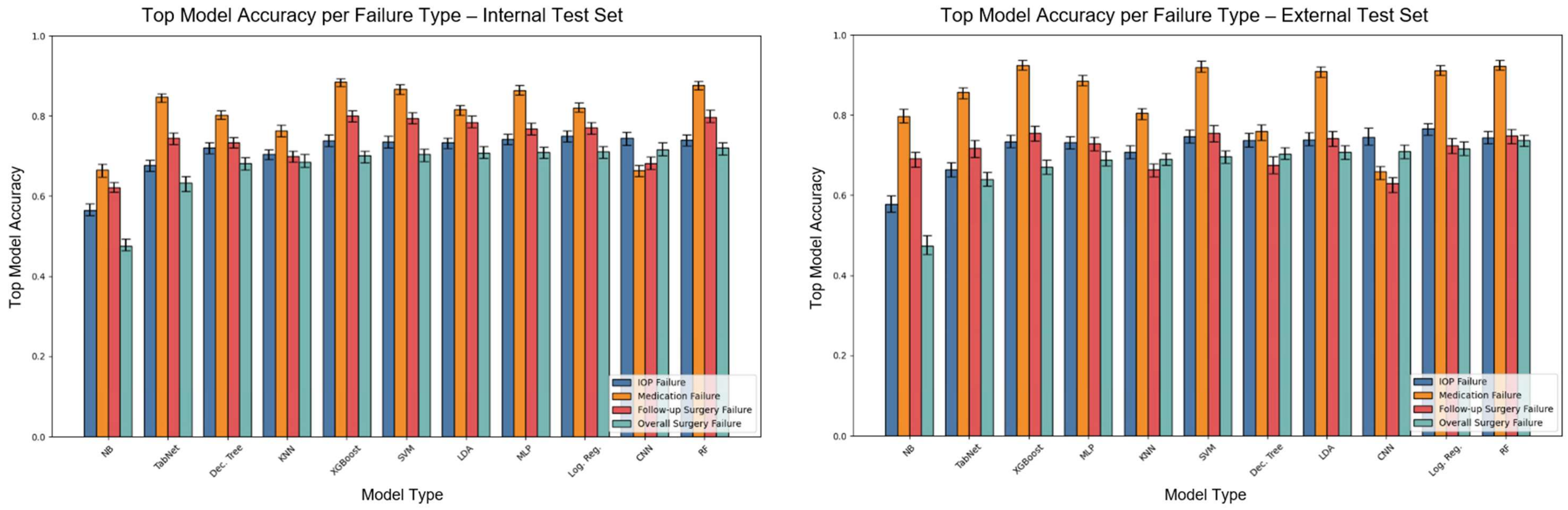

Supplementary Figure 3: Accuracy on the internal and external test sets for models predicting overall surgical failure and specific failure criteria.
The bars illustrate the test set AUROC for each model based on individual failure criteria, utilizing the optimal set of hyperparameters. Error bars indicate the 95% confidence intervals. The models included are Dec. Tree (Decision Tree), XGBoost (Gradient Boosting), KNN (K-Nearest Neighbors), LDA (Linear Discriminant Analysis), Log. Reg. (Logistic Regression), MLP (Multilayer Perceptron), NB (Gaussian Naïve Bayes), RF (Random Forest), SVM (Support Vector Machine), TabNet and 1D-CNN (1-Dimensional Convolutional Neural Network).
